# CD133 Expression and the Prognosis of Colorectal Cancer: A Systematic Review and Meta-Analysis

**DOI:** 10.1371/journal.pone.0056380

**Published:** 2013-02-11

**Authors:** Shicai Chen, Xinming Song, Zhihui Chen, Xinxin Li, Mingzhe Li, Haiying Liu, Jianchang Li

**Affiliations:** 1 Department of Gastrointestinal Tumor Surgery, Affiliated Tumor Hospital of Guangzhou Medical College, Guangzhou, China; 2 Department of Gastrointestinal and Pancreatic Surgery of the First Affiliated Hospital, Sun Yat-sen University, Guangzhou, China; Sapporo Medical University, Japan

## Abstract

**Objective:**

CD133 has recently been reported as a marker of cancer stem-like cells in colorectal cancer (CRC). However, its predictive value in CRC still remains controversial. In this study, we aimed to evaluate the association between the expression of CD133 and clinicopathological features and the outcome of CRC patients by performing a meta-analysis.

**Methods:**

A comprehensive literature search for relevant studies published up to December 2012 was performed using PubMed, MEDLINE and ISI Web of Science. Only articles in which CD133 antigen was detected in situ localisation by immunohistochemical staining were included. This meta-analysis was done using RevMan 4.2 software.

**Results:**

We found that a total of 15 studies involving 810 CD133-high and 1487 CD133-low patients met the inclusion criteria for the analysis of 5-year overall survival (OS) rate. In a random-effects model, the results showed that CD133-high expression in colorectal cancer was an independent prognostic marker correlating with both OS rate (RR = 0.67, 95%CI 0.54–0.82, P<0.01) and disease free survival (DFS) rate (RR = 0.71, 95%CI 0.52–0.96, P = 0.03). CD133-high expression was also associated with more T3,4 tumor invasion, N positive and vascular invasion cases, corresponding to a risk difference of 1.12 (95%CI 1.01–1.23, P = 0.03), 1.31 (95%CI 1.06–1.63, P = 0.01) and 1.24 (95%CI 1.08–1.41, P<0.01), respectively. However, when types of histology, lymphatic invasion and distant metastasis were considered, CD133 overexpression was not significantly related with these clinicopathological parameters.

**Conclusion:**

Our meta-analysis results suggest that CD133 is an efficient prognostic factor in CRC. Higher CD133 expression is significantly associated with poorer clinical outcome and some clinicopathological factors such as T category, N category and vascular invasion in CRC patients.

## Introduction

Although treatments for CRC have developed rapidly in recent years, CRC still remains the second most common cause of cancer-related death[1]. It has been reported that a rare subpopulation of cells with special surface markers within CRC possesses the potential to initiate and sustain tumour growth. These so-called cancer stem cells(CSCs) associated with tumour relapse and progression are considered to be responsible for the poor outcome of CRC. During the past few years, several cell surface markers have been identified as stem cell markers in colorectal cancer. Among these markers, CD133 is believed to be the most robust surface marker for CRC stem cells by now.

CD133 molecule (also known as prominin-1) is a five-transmembrane glycoproteins with a molecular weight of 120 kDa and it is shown to be mainly localized in membrane protrusions[2]. Nevertheless its biological function is still mysterious. CD133 was first discovered on normal human hematopoietic stem cells in 1997[3], [4]. Subsequently, isolation based upon CD133 positivity had revealed tumor-initiating cells in some neoplasms such as leukemia[4], brain cancer[5], ovarian cancer[6], hepatocellular carcinoma[7], prostate[8] and pancreas[9]. In 2007, two researchers respectively reported that CD133-positive cells separated from colorectal cancer exhibited the C-IC properties of self-renewal and high tumorigenic potential. O’Brien used renal capsule transplantation in immunodeficient NOD/SCID mice to identify a human colon cancer-initiating cell (CC-IC) [10]. She found that all CC-ICs were CD133+ and the CD133- cells that comprised the majority of the tumour were unable to initiate tumour growth; When calculated by limiting dilution analysis, there was one CC-IC in 5.7×10^4^ unfractionated tumour cells, whereas there was one CC-IC in 262 CD133+ cells. Ricci-Vitiani showed that tumorigenic cells in colon cancer were included in the high-density CD133+ population, which accounted for about 2.5% of the tumour cells[11].

It had been suggested that CD133+ tumour cells were more resistant to radiochemotherapy than CD133- cells in CRC[12], [13]. So it would be expected that the CSC burden in colorectal cancer is of relevance for patients’ outcome. First of all, Horst et al showed that CD133 expression in CRC was an independent prognostic marker that correlates with low survival[14]. However, other studies failed to demonstrate such an association between the presence of CD133+ cells and poor clinical outcome of CRC[15], [16]. Insufficient samples and some other factors have resulted in controversial results of different clinical studies. The present meta-analysis aims to determine the value of CD133 as a prognostic marker for CRC. Also the correlation between CD133 and several clinicopathological features of CRC would be examined in this study.

## Materials and Methods

### Search Strategy

We conducted a systematic literature search of following databases: PubMed from 1966 through December 2012, MEDLINE from 1966 through December 2012 and Web of Science from 1970 through December 2012. Search terms included “AC133 antigen ”, “CD133”, “prominin-1” and “colon cancer”, “rectal cancer”, “colorectal cancer”, combined with “Neoplastic Stem Cells(s)”, “cancer stem cell (s)” or “tumor-initiating cell (s)”. Only articles that detected CD133 in situ localisation by immunohistochemistry method were included in this study. The title and abstract of each study identified in the search was scanned to exclude any clearly irrelevant ones. The remaining articles were browsed to determine whether they contained information on the topic of interest. The reference lists of articles with information on the topic were also reviewed for additional pertinent studies.

### Selection Criteria

The studies included in this meta-analysis could be either randomized controlled studies (RCTs) or observational studies (case-control or cohort) that evaluated the association between CD133 expression and the risk of CRC. Articles were excluded from the analyses if there was insufficient published data for determining an estimate of RR and a CI, or if the full text couldn’t be found. If there were several publications from the same population, only the most recent reports were selected for analysis.

We did not intend to assess the methodologic quality of the primary studies, given that quality scoring in meta-analyses of observational studies is controversial[17], [18]. Scores constructed in this ad hoc fashion may lack demonstrated validity, and results may not be associated with quality[19].

### Data Extraction

All data were independently abstracted by two reviewers with standardized data abstraction tool. Disagreements in data extraction were resolved by consensus, referring back to the original article. The following data were sought from each article: first author’s last name, year of publication, country of the population studied, number of participants, staining patterns of CD133, the choice of cutoff scores for the definition of positive staining or staining intensity, duration of follow-up, T category, N category, distant metastasis, histology, lymphatic invasion, vascular invasion and most importantly including 5-year overall survival (OS) rate and 5-year disease free survival (DFS) rate.

Since the cutoff value for CD133-high group varied with different studies, here we defined CD133-high expression values according to the original articles. Staging of CRC was based on the UICC classification revised in 2009. Tumour differentiation was graded by a pathologist according to the World Health Organization (WHO) classification system. In order to avoid some studies contributing very long-term follow-up data as compared with others, both OS and DSF were standardized to include 5 years of follow-up in all studies. For those articles which didn’t provide 5-year OS rate and 5-year DFS rate directly, Kaplan–Meier curves were read using GetData Graph Digitizer 2.24(http://getdatagraph-digitizer.com).

### Statistical Analysis

The statistical process was performed according to the guidelines proposed by the Meta-Analysis of Observational Studies in Epidemiology group[20]. Relative risk (RR) with 95% confidence intervals(CIs) were calculated using Review Manager 4.2. Between-study heterogeneity was measured using the Q statistic (P<0.05 was considered of statistically significant heterogeneity). Fixed-effects models (Mantel-Haenszel) assume that differences between the results of various studies are due to chance. Random-effects models (DerSimonian and Laird) consider the results may differ genuinely between studies. In the absence of heterogeneity, both the fixed- and the random-effects models provide similar results. When heterogeneity is present, the random-effects model is considered to be more appropriate than a fixed-effects model, resulting in wider intervals and a more conservative estimate of treatment effect[21]. The potential for publication bias was assessed by using the Begg rank correlation method and the Egger weighted regression method (software stata11.0, P<0.05 was considered representatively of statistically significant publication bias) [22]. All P values above are two tailed.

## Results

### Search Results

**Figure 1:**
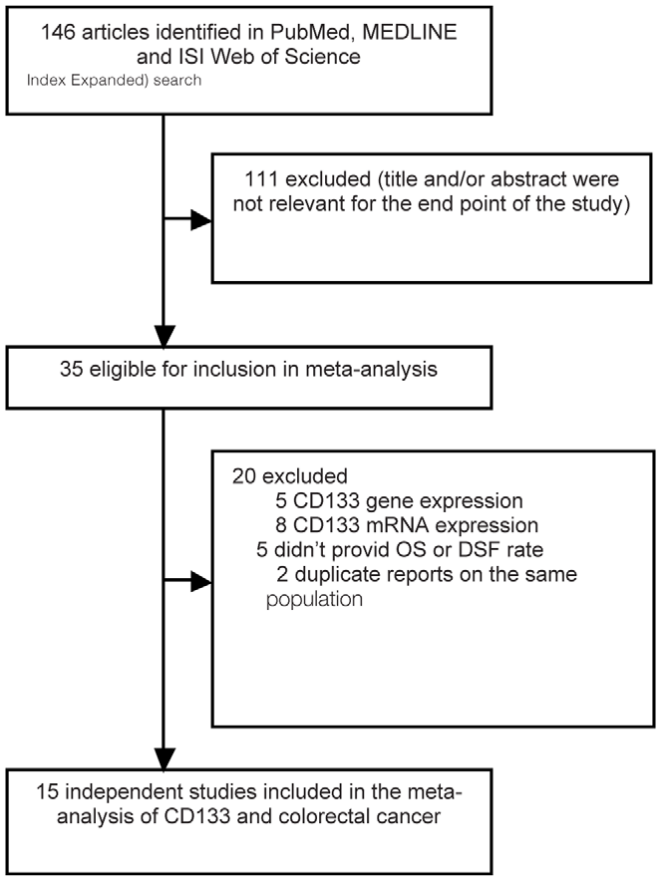
Flowchart of selection of studies for inclusion in meta-analysis.

Detailed search steps were described in Figure 1. The initial search algorithm retrieved a total of 146 studies according to the inclusion/exclusion criteria stated above. After titles and abstracts were previewed, only 35 identified studies concerning CD133 and the risk of CRC were further evaluated. Of the published studies, 20 reports were excluded: five were about CD133 gene expression[23]–[27], eight were about CD133 mRNA expression[28]–[35], another five didn’t provid OS or DSF rate[12], [13], [16], [36], [37] and the other two duplicate reports on the same population[14], [38]. Eventually, a total of 15 observational retrospective studies met the predefined inclusion criteria including about 2297 participants[15], [39]–[52]. All these studies evaluated the expression of CD133 and risk of colorectal cancer by immunohistochemical staining method(Table 1).

**Table 1:** **Characteristics of the included studies.**

Author	Year	Country	Number of Patients	Duration of Follow-up	Staining patterns	Cutoff scores (high/low)	T category (T1,2/T3,4)	N category (Negative/ Positive)	Distant metastasis (M0/M1)	Histology (Well,mod/ Por)	Lymphatic Invasion (N/Y)	Vascular Invasion (N/Y)	5-year OS rate	5-year DFS rate
Kojima[39]	2008	Japan	189	Median 2024 days	membrane	>10% (21/168)	H(2/19) L(39/129)	H(9/12) L(91/77)	H(16/5) L(147/21)	H(21/0) L(139/29)	H(10/11) L(96/72)	H(2/19) L(40/128)	H 61.8%(13/21) L 81.3%(113/139) *	H 68.7%(11/16) L 78.5%(97/124)*
Wang[40]	2009	China	73	>3 years	membrane	>10% (38/35)	H(14/24) L(23/12)	NA	NA	H(22/16) L(22/13)	H(9/29) L(2/33)	NA	H 60.1%(23/38) L 91.6%(32/35) *	H 42.6%(16/38) L 91.2%(32/35)*
Li[41]	2009	China	104	5 years	membrane	>5% (42/62)	NA	NA	NA	NA	NA	NA	H 45.2%(19/42) L 77.4%(48/62)	NA
Horst[42]	2009	Germany	110	median 7.9 years	membrane	>50% (36/74)	H(8/28) L(31/43)	NA	NA	H(36/0) L(74/0)	NA	NA	H 44.6%(16/36) L 74.3%(55/74)*	NA
Takahashi[43]	2010	Japan	151	Median 41 months	Membrane and cytoplasm	>50% (41/110)	H(12/29) L(26/84)	H(21/20) L(58/52)	H(30/11) L(84/26)	NA	H(11/30) L(27/83)	H(18/23) L(52/58)	H 45.5%(19/41) L 74.3%(82/110)	H 61.9%(19/30) L 76.7%(64/84)
Kojima(2) [44]	2010	Japan	43	NA	membrane	>10% (8/35)	H(3/5) L(18/17)	H(4/4) L(26/9)	NA	NA	H(4/4) L(22/13)	NA	H 72.5%(6/8) L 96.5%(34/35)*	H 22.8%(2/8) L 73.1%(26/35)*
Xi[45]	2011	China	201	>5 years	Membrane and cytoplasm	score ≥5 (110/91)	H(23/87) L(52/39)	H(53/57) L(75/16)	H(100/10) L(90/1)	H(101/9) L(76/15)	NA	NA	H 22.7%(25/110) L 82.4%(75/91)	NA
Choi[15]	2009	South Korea	523	NA	cytoplasm	>0% (128/395)	H(9/119) L(49/346)	H(56/72) L(178/217)	H(124/4) L(378/17)	H(100/28) L(316/79)	H(56/72) L(169/226)	H(126/2) L(387/8)	H 70.8%(90/128) L 68.0%(269/395)*	NA
Garcia[46]	2010	Spain	45	Median 40 months	membrane	>10% (17/28)	NA	NA	NA	NA	NA	NA	H 92.0%(15/17) L 64.0%(18/28)*	H 64.9%(11/17) L 34.1%(10/28)*
Nagata[47]	2011	Japan	27	NA	Membrane	>0% (16/11)	NA	NA	NA	NA	NA	NA	H 13.3%(2/16) L 20.0%(2/11)	NA
Zhang[48]	2012	China	125	Median 6.5 years	membrane	score ≥4 (45/80)	H(4/41) L(12/68)	H(18/27) L(43/37)	NA	H(41/4) L(75/5)	NA	NA	H 57.8%(26/45) L 75.0%(60/80)	NA
Li(2) [49]	2012	China	200	1 to 155 months	membrane and cytoplasm	Scores ≥4 (84/116)	H(6/78) L(17/99)	H(35/49) L(78/38)	NA	H(58/26) L(96/20)	NA	NA	H 31.0% (26/84) L 94.8% (110/116)	NA
Coco[50]	2012	Italy	137	NA	membrane and cytoplasm	>5% (65/72)	H(19/46) L(10/62)	H(34/31) L(42/30)	NA	H(41/24) L(54/18)	NA	NA	H52%(34/65) L72.2%(52/72) *	H47.8%(31/65) L71.8%(52/72) *
Hongo[51]	2012	Japan	303	NA	membrane	>5% (140/163)	H(38/102) L(45/118)	H(94/46) L(106/57)	NA	H(137/3) L(156/7)	H(112/28) L(131/32)	H(49/91) L(77/86)	H85%(119/140) L96.3%(157/163) *	NA
Bonetti[52]	2012	Italy	95	Median 108 months	membrane and cytoplasm	>50% (19/76)	NA	NA	NA	NA	NA	H(11/8) L(68/8)	H47%(9/19) L91.8%(70/76) *	H36.6%(7/19) L85.3%(65/76) *

H: high expression; L: low expression; NA:not available; *:data read by GetData Graph Digitizer.

### Characteristics of eligible Studies

All 15 eligible studies were listed in Table 1. Five reports originated from Japan, five from China, one from Germany, one from South Korea, one from Spain and two from Italy. Thirteen reports used whole tissue sections for immunohistochemical analyses and only two utilized tissue microarray[15], [46]. Since Horst had compared serial tumour sections stained with three different antibodies raised against CD133 and all three antibodies revealed comparable staining patterns of positively and negatively stained tumour cells[14]. So here, choice of antibody was not taken into account as a confounding factor affecting the outcome of this study.

The Kojima’s studies included two different patient cohorts[39], [44]. First, a series of 189 CRC patients were treated surgically(155 curative, 34 palliative) and none of the patients received preoperative adjuvant therapy, but all the stage III and IV patients received postoperative adjuvant chemotherapy[39]. Second, 43 rectal cancer patients who received preoperative chemoradiation therapy (CRT) was included and after operation, all patients with pathological stage III disease received postoperative 5-fluorouracil-based chemotherapy[44]. In Wang’s study, 73 rectal cancer patients were treated with preoperative radiation before TME[40]. In another study, 104 colon carcinoma patients with TNM stage IIIB were subject to radical resection. None of the cases had undergone preoperative CRT and all of them were subject to 5-Fu based postoperatively adjuvant chemotherapy for six months[41]. Moreover in Horst’s study, none of the 110 CRC patients received preoperative or postoperative adjuvant therapy[42]. 151 patients who underwent surgical treatment for CRC were enrolled in Takahashi’s study and none of the patients received any preoperative therapy, but most of the stage II–IV patients received adjuvant chemotherapy[43]. Xi’s study contained a total of 201 consecutive CRC patients who underwent curative surgical resection[45]. In addition, Choi’s study consisted of a consecutive series of 523 colorectal adenocarcinomas[15]. And Garcia’s study included 45 CRC patients treated with preoperative CRT and all had intermediate responders (TRG 2 and 3) [46]. 27 cases in which surgery was performed for resection of local recurrent lesions in the pelvis were included in Nagata’s report[47]. In Zhang’s study, 125 stage II or III colon cancer specimens without neoadjuvant chemoradiotherapy were obtained after radical resection. 90 patients received postoperative Fu-based adjuvant chemotherapy and 35 stage II patients did not receive adjuvant interventions[48]. Li’s study included 200 colorectal adenocarcinomas and adjuvant chemoradiotherapy was not provided[49]. 137 patients who had undergone curative surgery for colon cancer were selected for Coco’s study[50]. Hongo et al. showed that a total of 303 colorectal cancer patients were involved for evaluating CD133 expression, respectively 225 patients who underwent curative resection and 78 patients who received preoperative chemoradiotherapy and curative resection^51^. 95 patients with stage I colorectal adenocarcinoma were selected in Bonetti’s study^52^.

### Correlation of CD133 with clinicopathological Parameters

The association between CD133 and several clinicopathological parameters was illustrated in Figure S1, S2, S3, S4, S5, S6. The expression of CD133 correlated with more T3,4 category patients (pooled RR  =  1.12, 95%CI 1.01–1.23, P = 0.03 random-effect). Moreover, CD133-high expression was also associated with more N positive and vascular invasion cases, leading to a risk difference of 1.31 (95%CI 1.06–1.63, P = 0.01 random-effect) and 1.24 (95%CI 1.08–1.41, P<0.01 fixed-effect), respectively. However, CD133 was not associated with other clinicopathological features such as histology (pooled RR  =  1.00, 95% CI 0.92–1.08, P  =  0.98 random-effect), lymphatic invasion (pooled RR  =  0.98, 95% CI 0.88–1.10, P  =  0.78 fixed-effect) or distant metastasis (pooled RR  =  1.42, 95% CI 0.92–2.19, P  =  0.11 fixed-effect).

### Impact of CD133 on 5-year OS and DSF Rate of CRC

**Figure 2:**
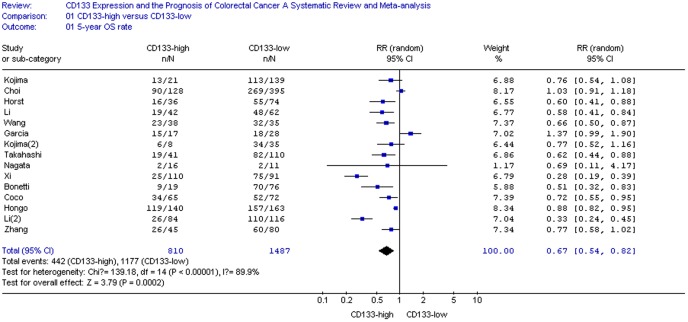
CD133 expression and 5-year OS rate.

**Figure 3:**
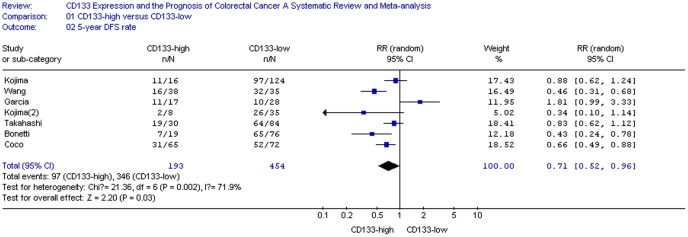
CD133 expression and 5-year DFS rate.

The relationship between CD133 expression and the risk of CRC was illustrated in Figure 2–3. Fifteen reports including a total of 2297 patients in regard to the association of CD133 and 5-year OS rate could be obtained from published information, whereas for 5 studies (647 patients in all) information for the correlation of CD133 with 5-year DSF rate could be extracted from the published articles (Table 1). CD133 overexpression was statistically significantly associated with poor 5-year OS rate in either a random-effects model (Figure 2. RR = 0.67, 95% CI = 0.54–0.82, P <0.01) or a fixed-effects model (data not showed). 5-year OS rate was 0.67-fold lower in CD133-positive patients(P<0.01). Furthermore, slight difference had been found in 5-year DSF rate between CD133-high and CD133-low groups assuming a random-effects model (Figure 3. RR = 0.71, 95% CI = 0.52–0.96, P  = 0.03).

### Sensitivity Analyses

We also performed sensitivity analyses in order to further explain the results regarding of OS. Samples (whole tissue sections versus tissue microarray), immunohistochemical staining patterns (membrane versus cytoplasm) and different cutoff scores were included as factors in sensitivity analyses.

The results were shown in Table 2. Patients with CD133-high expression base on the whole tissue sections seemed to have worse 5-year OS rate than those with CD133-low expression(RR = 0.60, 95% CI = 0.46–0.78, P<0.01). Nevertheless, the event did not show any significant difference between the CD133-high and CD133-low subgroups when immunohistochemisty was carried out using tissue microarray(RR = 1.06, 95% CI = 0.94–1.20, P = 0.32). In 14 reports with membranous or combining with cytoplasmic CD133 staining, 5-year OS rate was poorer in CD133-high expression group. However, no difference had been found in patients with cytoplasmic CD133 staining only. When the cutoff scores were considered, the results varied with the change of cutoff level definitions. Two articles (>0%) and another four (>10%) did not demonstrate any significant difference, while the other nine articles (3>5%, 3>50% and 3 scores≥4, respectively) displayed significant difference between the CD133-high and CD133-low patients.

**Table 2:** **Results of sensitivity analyses.**

Categories	Studies (n)	RR(95% CI)	P value	Effect model
Samples				
Whole tissue sections	13	0.60(0.46–0.78)	<0.01	Random
Tissue microarray	2	1.06(0.94–1.20)	0.32	Fixed
Staining patterns				
Membrane	9	0.76(0.59–0.98)	<0.01	Random
Cytoplasm	1	1.11(0.72–1.71)	0.64	Fixed
Membrane and Cytoplasm	5	0.46(0.31–0.69)	<0.01	Random
Cutoff scores				
>0%	2	1.03(0.90–1.17)	0.70	Fixed
>5%	3	0.74(0.55–1.00)	0.05	Random
>10%	4	0.85(0.61–1.20)	0.36	Random
>50%	3	0.59(0.47–0.74)	<0.01	Fixed
Scores≥4	3	0.41(0.21–0.81)	0.01	Random

### Publication Bias

**Figure 4:**
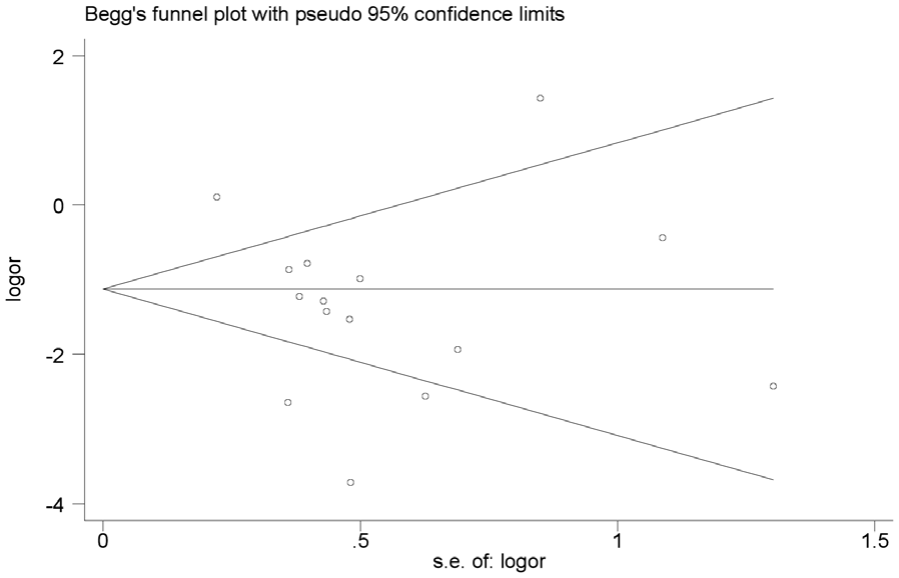
Begg’s test result of 5-year OS rate.

**Figure 5:**
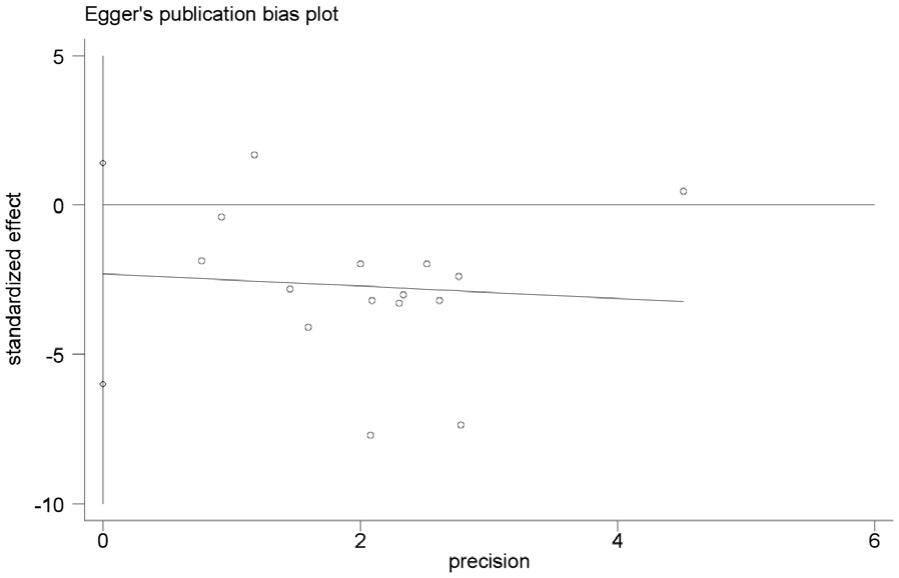
Egger’s test result of 5-year OS rate.

**Figure 6:**
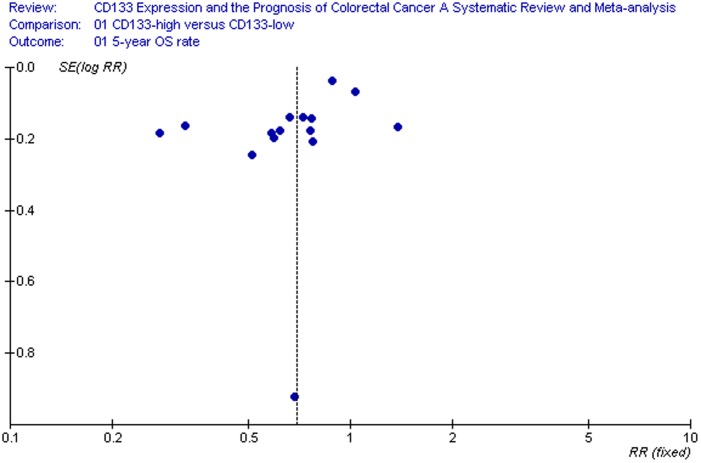
Funnel plot of studies used in the analysis of CRC 5-year OS rat

We performed an analysis to evaluate the influence of individual studies on the summary 5-year OS rate. The effect was not dominated by any single study, and omission of any study at a time made no difference(Begg’s P value = 0.373 and Egger’s P value = 0.202, Figure 4, 5). In addition, funnel plot was performed to estimate the publication bias of the included literature. The shapes of the funnel plots showed that selected studies did not have apparent asymmetry (Figure 6).

## Discussion

To our knowledge, this meta-analysis is the first study which systematically estimates the association between CD133 expression and the risk of CRC and its clinicopathological parameters. Yang’s report was mainly about the value of CD133 as a biomarker of CSCs for CRC and brain tumors, and the outcome measures were to assess colony formation rate and xenotransplanted tumor formation rate[53]. In our study, however, we are going to evaluate the expression of CD133 and its prognostic value for CRC patients. Although Shmelkov et al had showed that CD133 expression was not restricted to CSCs of colorectal cancer and CD133- subsets could also initiate tumors[54], This present results indicate that CD133, as detected with immunohistochemistry method, is significantly associated with worse 5-year OS rate and DSF rate in CRC patients. Besides, CD133-high expression was also related with more T3,4 category, N positive and vascular invasion patients. Up to now, CEA and CA 19–9 are the most widely applied markers in gastrointestinal malignancies and elevated levels of both CEA and CA19–9 have also been suggested to be associated with poor prognosis in CRC[55], [56]. However, because of their low sensitivity, their secretion rates from individual tumors and nonspecific elevations reduce their tumor marker utility and indicate the need for additional more reliable markers for CRC[57]. Recently, CD133 is considered to be the most useful surface marker for CRC stem cells and its major prognostic impact in CRC patients have been indicated based on our conclusion.

However, results should be interpreted very cautiously. Several reasons that may influence the conclusion of each included report need to be considered: sample size, methodology (tissue microarray versus whole tissue sections), immunohistochemical staining patterns(membrane versus cytoplasm), and the choice of cutoff scores for the definition of positive staining or staining intensity[16].

Garcia et al. had evaluated CD133 antigen using tissue microarrays(TMA) and meanwhile confirmed this marker in the whole section[46]. No differences had been found for CD133 expression in TMA though the prognostic results were close to statistical significance. Also, when examining CD133 expression in the whole section, there was a moderate correlation with TMA and the prognostic significance was lost. It is possible that the false-negative cases obtained in TMA biased the result towards a worst prognosis for negative cases. Another reason may be that CD133 immunohistochemistry is not pinpointing tumour initiating cells, but instead some other related pathway status[58]. Moreover, CD133 is mainly expressed in well- and moderately-differentiated adenocarcinomas, and CD133 negativity may reflect tumour ‘budding’, a more undifferentiated state at the front of invasion where tumour cells are known to almost always be CD133 negative[14], [59]. In our study, combined analyses of 13 included reports detecting CD133 antigen in whole tissue sections indicated a poor 5-year OS outcome in CD133-high expression patients, while the result of two reports detecting CD133 by TMA showed no significant difference in 5-year OS rate between the CD133-high and CD133-low groups.

Two patterns of CD133 expression by immunohistochemical method had been found in CRC specimens. Some studies showed that the CD133 antigen was exclusively on the cell membrane at the luminal surface of cancer gland[39]–[42], [44], [46]–[48], [51]. Nevertheless, others demonstrated that CD133 could be detected both on membrane and cytoplasm in CRC[43], [45], [49], [50], [52], as well as in pancreatic cancer[60]. Only one selected literature displayed cytoplasmic CD133 staining[15]. In Takahashi’s study, the pattern of CD133 expression in CRC tumor cells was divided into ‘membranous expression’ and ‘cytoplasmic expression’ [43]. Immunolocalization of CD133 reflected different clinical significance. Membranous CD133 overexpression correlated with patient survival, recurrence-free survival and chemoresistance, Nevertheless, cytoplasmic expression was not an independent marker for patient survival and disease recurrence. It was speculated that CD133 shift from cytoplasmic localization to membranous localization, displaying the transition of epithelial cells to a more invasive phenotype. The potential mechanism might be opposite to that of CD24 presumed by Weichert et al [61]. This present result suggested that CD133 overexpression locating on membrane but not on cytoplasm was probably a useful marker to predict clinical outcome of CRC patients.

The cutoff scores for defining CD133 overexpression have not been unified between studies so far. The results may change according to the cutoff value. It seemed that a higher level of cutoff score, 50% for example, would be more helpful leading to a differential conclusion.

Besides CD133, some other cell surface molecules such as CD44, CD24, CD166 and EpCAM have been considered as putative CSC markers in CRC. And the combination of these markers may provide a better selection of CSCs[62]. As for the prognostic value of these markers, Horst et al implied that CD133 might be of the most clinical relevance, while the combined evaluation of CD133, CD44, and CD166 might even be more valuable to separate high-risk from low-risk colorectal cancer cases[42].

Other than displaying CD133 molecule in situ by immunohistochemical staining, some studies have examined CD133 gene or mRNA expression using reverse transcriptase-polymerase chain reaction(RT-PCR) method. Elevated CD133 gene level may predict distant recurrence and poor prognosis of patients with CRC[23]–[27] ^.^. Lin used semiquantitative real-time RT-PCR to quantify CD133 mRNA levels in peripheral blood mononuclear cells from patients with colon cancer and he found that elevated CD133 mRNA levels at ≥4.79 predicted colon cancer recurrence independent of TNM stage IV disease[28]. Artells assessed CD133 mRNA expression levels by RT-QPCR in tumour and matched normal tissue from 64 stages I–III CRC patients and observed longer relapse-free interval and OS in patients with lower levels of CD133[29]. Huh also demonstrated elevated CD133 mRNA levels might represent more aggressive tumor biology and poorer survival in patients with CRC, correlating with a high level of MSI status[30]. Nakamura indicated that CD133/CEA/CK20 mRNAs in peritoneal washings were independent prognostic factors for OS and PFS[31]. Iinuma showed that in patients with Dukes’ stage B and C CRC who required adjuvant chemotherapy, detection of CEA/CK/CD133 mRNA in peripheral blood was a useful tool for determining which patients were at high risk for recurrence and poor prognosis[32].

Our results should be interpreted cautiously since some limitations exist in this present meta-analysis. First of all, no RCTs had been found and the number of included studies was relatively small with only about 2300 cases. CRC patients had received different treatments (perioperative adjuvant therapy or just curative surgical resection); preoperative TNM category and histologic types were various. Whereas, we were unable to assess these potential confounders present in individual studies. Second, although we tried to identify all relevant data, potential publication bias was unavoidable and some data could still be missing. Lugli’s study did not provide survival time associated with CD133[16]. This missing information reflected ‘‘negative’’ or more conservative association of CD133 with survival that could reduce the significance of CD133 expression as a predictor of OS. Third, although immunohistochemistry was the most commonly applied method for detecting CD133 in situ, RT-PCR method had also been used for the evaluation of the levels of CD133 gene or mRNA expression in tumour tissue, peritoneal washings and peripheral blood. Studies measuring CD133 gene or mRNA level by RT-PCR was not yet included in this meta-analysis. Moreover, the cutoff value was defined differently (0%, 5%, 10% or 50%) in these studies, leading to between-study heterogeneity. Thus we had adopted random effect model and subgroup sensitivity analyses to adjust for the shortcomings. Finally, except for CD133, this present study didn’t examine the correlation between other putative CSC markers and the risk of CRC.

In summary, despite the limitations listed above, this present study shows a significant correlation between CD133 expression and 5-year OS rate as well as DFS rate in CRC patients. CD133 may have prognostic significance for patients with CRC based on currently obtained data. Further studies using additional putative CSC surface markers in combination with CD133 are required to evaluate their potential use in predicting patients’ outcome.

## Supporting Information

Figure S1 **CD133 expression and T category.**
(DOC)

Figure S2 **CD133 expression and N category.**
(DOC)

Figure S3 **CD133 expression and vascular invasion.**
(DOC)

Figure S4 **CD133 expression and histologic types.**
(DOC)

Figure S5 **CD133 expression and lymphatic invasion.**
(DOC)

Figure S6 **CD133 expression and distant metastasis.**
(DOC)
