# Supplementary figures and images for: CD133 Expression and the Prognosis of Colorectal Cancer: A Systematic Review and Meta-Analysis
Source: PLoS One. 2013 Feb 11;8(2):e56380. doi: 10.1371/journal.pone.0056380 (PMC3569427; doi:10.1371/journal.pone.0056380)

**
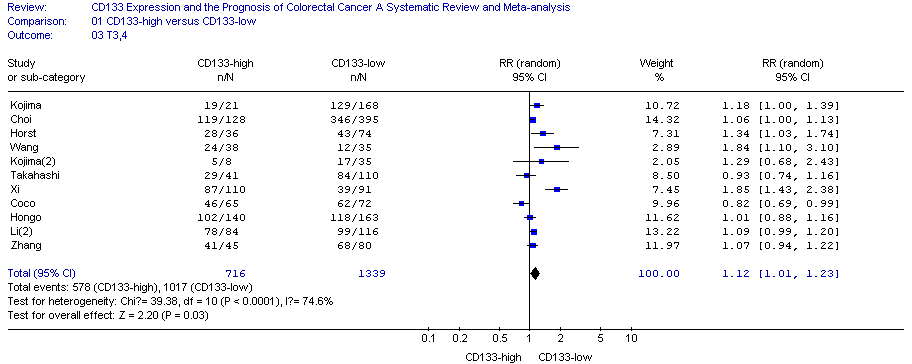
**

Fig. S1. CD133 expression and T category.

Supplement: Figure S1 — CD133 expression and T category. (DOC) [file pone.0056380.s001.doc]

**
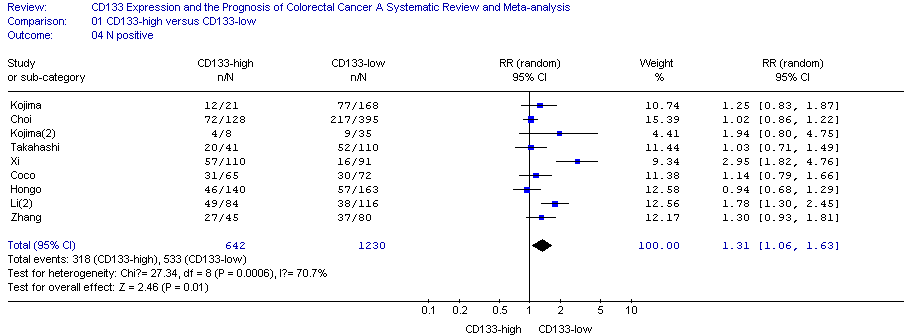
**

Fig. S2. CD133 expression and N category.

Supplement: Figure S2 — CD133 expression and N category. (DOC) [file pone.0056380.s002.doc]

**
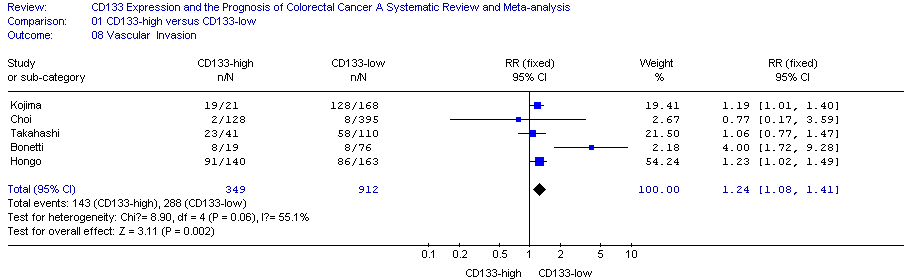
**

Fig. S3. CD133 expression and vascular invasion.

Supplement: Figure S3 — CD133 expression and vascular invasion. (DOC) [file pone.0056380.s003.doc]

**
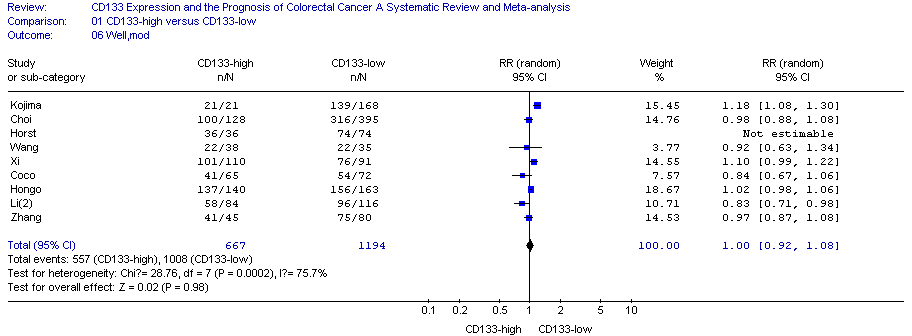
**

Fig. S4. CD133 expression and histologic types.

Supplement: Figure S4 — CD133 expression and histologic types. (DOC) [file pone.0056380.s004.doc]

**
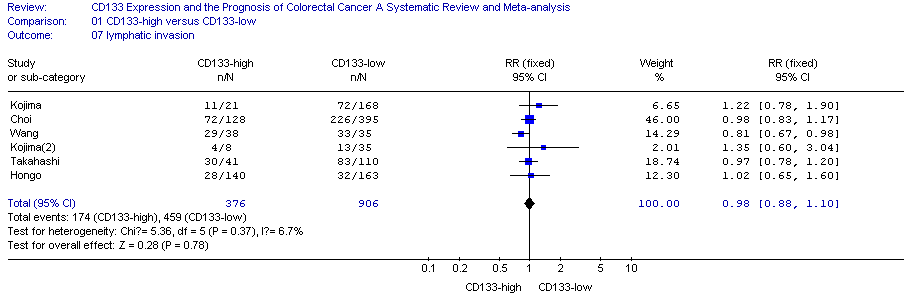
**

Fig. S5. CD133 expression and lymphatic invasion.

Supplement: Figure S5 — CD133 expression and lymphatic invasion. (DOC) [file pone.0056380.s005.doc]

**
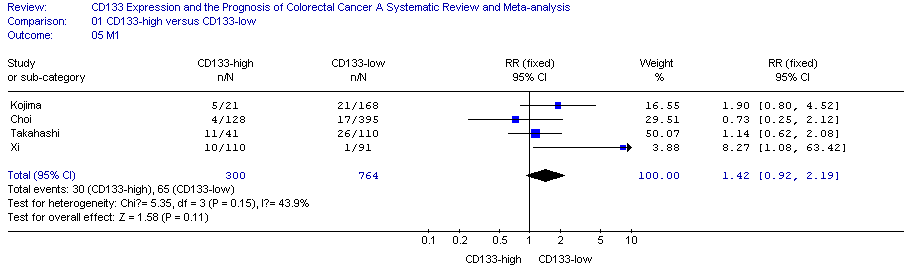
**

Fig. S6. CD133 expression and distant metastasis.

Supplement: Figure S6 — CD133 expression and distant metastasis. (DOC) [file pone.0056380.s006.doc]
